# ${\mathrm{MnBr}}_{2}$ on the Graphene on Ir(110) Substrate: Growth, Structure, and Super‐Moiré

**DOI:** 10.1002/smll.202509471

**Published:** 2026-01-05

**Authors:** Affan Safeer, Oktay Güleryüz, Nicolae Atodiresei, Wouter Jolie, Thomas Michely, Jeison Fischer

**Affiliations:** ^1^ II. Physikalisches Institut Universität zu Köln Zülpicher Straße 77 Köln Germany; ^2^ Peter Grünberg Institut (PGI‐1) Forschungszentrum Jülich Wilhelm‐Johnen‐Straße Jülich Germany

**Keywords:** super‐moiré, moiré, of moiré, manganese bromide, transition metal dihalides, ${\mathrm{MnBr}}_{2}$, 2D magnetic material

## Abstract

Single‐layer ${\mathrm{MnBr}}_{2}$ is grown on graphene (Gr) supported by Ir(110) and investigated using low‐energy electron diffraction, scanning tunneling microscopy, and spectroscopy. The structure and epitaxial relationship with the substrate are systematically characterized. The growth morphology strongly depends on the growth temperature, evolving from fractal to dendritic and eventually to compact dendritic–skeletal islands, reflecting changes in the underlying surface diffusion processes. The pronounced variation in the apparent height with tunneling conditions for the magnetic insulator is explained based on the measured electronic density of states. ${\mathrm{MnBr}}_{2}$ on Gr/Ir(110) constitutes a three‐lattice system, giving rise to a super‐moiré pattern – a moiré of moirés. The super‐moiré of ${\mathrm{MnBr}}_{2}$/Gr/Ir(110) is unique, as it involves a virtual moiré of ${\mathrm{MnBr}}_{2}$ with the Ir(110) surface lattice – two lattices not in contact with each other. Using a careful Fourier analysis, the known properties of Gr/Ir(110), and the results of ab initio calculations, the origin of the virtual moiré is uncovered and related to the inhomogeneous binding of Gr to Ir(110). Comparative experiments with ${\mathrm{MnBr}}_{2}$ on Gr/Ir(111) show similar growth and structure, but highlight the unique properties of the ${\mathrm{MnBr}}_{2}$/Gr/Ir(110) super‐moiré.

## Graphical abstract

The study realizes single‐layer ${\mathrm{MnBr}}_{2}$ via molecular beam epitaxy on graphene on Ir(110), unveiling a unique super‐moiré (“moiré of moirés”) formed by lattices of different symmetries. Remarkably, this super‐moiré is driven by a virtual interaction between non‐contacting layers. Together, these findings demonstrate that molecular beam epitaxy enables the creation of complex quantum architectures that cannot be achieved through conventional stacking of exfoliated layers.

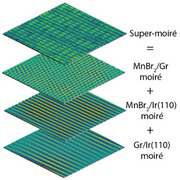

## 1 Introduction

Transition metal dihalides (TMDHs) are a class of materials that often exhibit magnetic ordering in their single‐layer form owing to their unique electronic structures and strong spin‐orbit coupling [1, 2, 3, 4, 5, 6]. Since the experimental discovery of intrinsic magnetic ordering in 2D materials, single‐layer TMDHs have attracted substantial research interest due to their potential as low‐dimensional magnets and their applicability in spintronics and quantum technologies [7, 8, 9]. Beyond their intriguing magnetic properties, epitaxially grown single‐layer TMDHs on graphene (Gr) undergo spontaneous self‐trapping of charges, forming quasiparticles with extremely long lifetimes, identified as polarons [10, 11, 12, 13, 14, 15, 16]. Because these quasiparticles are observed in epitaxially grown single‐layer TMDHs, understanding their exact nature requires a detailed analysis of the TMDH‐Gr epitaxial relationship.

The investigation of single‐layer TMDHs is often limited to first‐principles calculations, with few experimental validations. An example lacking experimental realization is 2D manganese dibromide (${\mathrm{MnBr}}_{2}$). Bulk ${\mathrm{MnBr}}_{2}$ crystallizes in a layered trigonal ${\mathrm{CdI}}_{2}$‐type structure (space group: $P\bar{3}m1$), where the layers are separated by van der Waals (vdW) gaps. In each layer, ${\mathrm{Mn}}^{2+}$ ions occupy an octahedral site coordinated by six ${\mathrm{Br}}^{-}$ ions in an edge‐sharing arrangement. At low temperatures, for bulk ${\mathrm{MnBr}}_{2}$ a double‐row striped antiferromagnetic ordering in the layers was measured [17, 18, 19, 20].

Single‐layer ${\mathrm{MnBr}}_{2}$ has been investigated theoretically using density functional theory (DFT) [3, 21, 22, 23 ]. The different calculations agree that single layers are stable in the trigonal phase, similar to the structure of a single layer in bulk crystals. According to calculations, antiferromagnetic order is also present in the single‐layer. The predicted bandgap of the magnetic insulator ranges from 2.61 [3] to 4.21 eV [21].

The preparation of single‐layer ${\mathrm{MnBr}}_{2}$ has not yet been realized. Preparation by exfoliation may be challenging because the strength of the in‐plane bonds of TMDHs is weaker than that of materials such as Gr or transition metal dichalcogenides. Presumably for this reason, most experimental studies on single layers of TMDHs have been conducted using samples grown by molecular beam epitaxy (MBE) [10, 11, 25, 26, 27, 28, 29, 30, 31, 32, 33, 34, 35]. Therefore, MBE of ${\mathrm{MnBr}}_{2}$ is also the method of choice used in this study.

In recent years, high‐order moiré patterns that emerge from the interference of more than two lattices, so‐called super‐moiré or moiré of moiré patterns, have captured significant research interest within the condensed matter physics community because they provide an exceptional platform for realizing exotic quantum states [34, 35, 36, 37, 38, 39, 40, 41, 42, 43, 44, 45]. For instance, super‐moiré systems can host topological flat bands [44, 45, 46, 47, 48] that enhance the electron–electron interactions, leading to the emergence of strongly correlated states, such as unconventional superconductors [49], fractional Chern insulators [50], quantum spin liquids [51], and Wigner crystals [52]. In k‐space, a moiré results from an integer linear combination of vectors $\vec{k}\left[A\right]$ and $\vec{k}\left[B\right]$ belonging to two different lattices A and B, resulting in moiré vectors $\vec{k}\left[A/B\right]$. Consequently, a super‐moiré is considered an integer linear combination of two different sets of moiré vectors that originate from two different moirés [38, 39]. This requires a minimal set of three lattices, A, B, and C, with sets $\vec{k}\left[A/B\right]$ and $\vec{k}\left[B/C\right]$. To date, super‐moiré systems have been mostly limited to twisted triple‐layer Gr [37, 42, 43, 44] or heterostructures between Gr and hexagonal boron nitride [35, 38, 39, 40, 41], where all the lattices have hexagonal symmetries.

Here, we present a comprehensive characterization of single‐layer ${\mathrm{MnBr}}_{2}$, providing a foundation for future studies of its magnetic and polaronic properties. The investigation includes the determination of the structure and layer stacking, the analysis of growth or annealing temperature‐dependent morphology linked to the underlying nucleation and diffusion processes, a systematic investigation of tunneling parameter‐dependent layer height and its link to the electronic structure, and lastly, the determination of the super‐moiré formed by the ${\mathrm{MnBr}}_{2}$ layer with the substrate moiré.

The case of ${\mathrm{MnBr}}_{2}$/Gr/Ir(110) expands the concept and knowledge of super‐moirés. Two of the three lattices are hexagonal (Gr and ${\mathrm{MnBr}}_{2}$), but one is rectangular, namely the surface lattice of Ir(110). The resulting super‐moiré not only exhibits intriguing complexity, but is also formed differently than anticipated previously. Instead of resulting from the combination of moiré vectors $\vec{k}\left[A/B\right]$ and $\vec{k}\left[B/C\right]$, its dominating Fourier coefficient results from a vector $\vec{k}\left[A/C\right]$, that is, in the present case $\vec{k}\left[{\mathrm{MnBr}}_{2}/\mathrm{Ir}\left(110\right)\right]$. This is surprising since Ir(110) and the ${\mathrm{MnBr}}_{2}$ layer are not in contact with each other. Hence, the effect is coined as virtual moiré. The presence of a virtual moiré in super‐moiré formation is also unique, as highlighted by comparative experiments for ${\mathrm{MnBr}}_{2}$ on Gr/Ir(111), which otherwise show a rather similar growth and structure of ${\mathrm{MnBr}}_{2}$.

## 2 Results and Discussion

### 2.1 Structure

**FIGURE 1:**
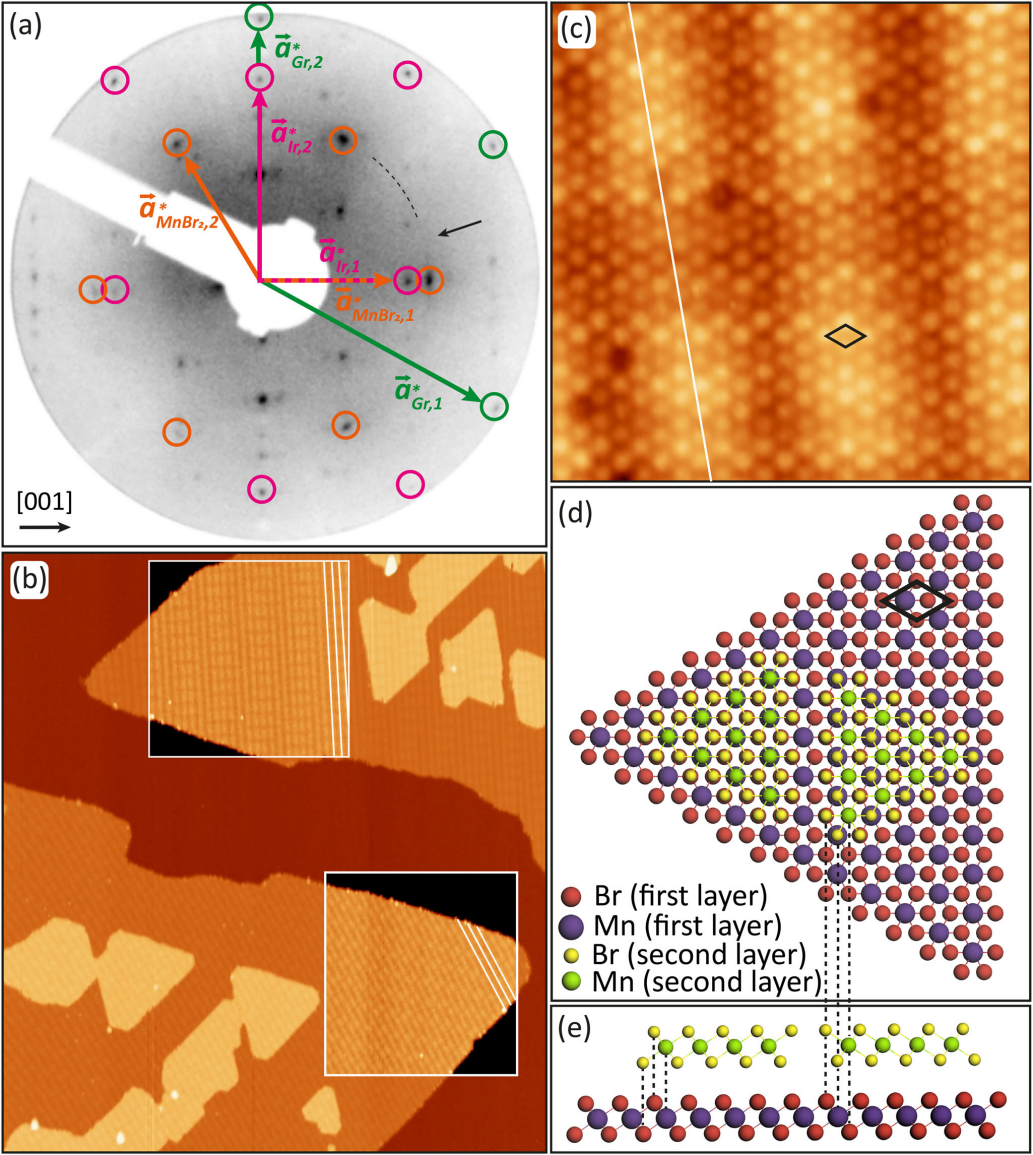
Structural characterization of ${\mathrm{MnBr}}_{2}$. (a) Contrast‐inverted 90 eV LEED pattern of ${\mathrm{MnBr}}_{2}$ after the growth of 1.1 ML at 400 K on Gr/Ir(110). First‐order Ir, Gr, and ${\mathrm{MnBr}}_{2}$ reflections are encircled in magenta, green, and orange, respectively. Reciprocal Ir, Gr, and ${\mathrm{MnBr}}_{2}$ primitive translations are indicated. (b) STM topography corresponding to the sample in (a). Contrast enhanced insets display striped moirés highlighted by white lines. (c) Atomically resolved STM image of ${\mathrm{MnBr}}_{2}$. White line highlights moiré stripe, as in (b). (d) Top and (e) side view ball models of the structure and bilayer stackings of ${\mathrm{MnBr}}_{2}$. All subfigures are oriented as indicated in (a), with the [001]‐direction of Ir being horizontal. ${\mathrm{MnBr}}_{2}$ unit cells are indicated as black rhombuses in (c) and (d). STM images are obtained at 300 K with (b) $V_{b}$ = −2 V, 50 pA and (c) $V_{b}$ = −2 V, 500 pA. Image sizes: (b) 180 nm × 200 nm, and (c) 7.5 nm × 7.5 nm.

The first insight into the ${\mathrm{MnBr}}_{2}$ structure and epitaxial relationship with Gr/Ir(110) is provided through the low energy electron diffraction (LEED) pattern in Figure 1a, taken after the growth of 1.1 ML at 400 K. The LEED pattern displays the first‐order diffraction spots of the rectangular lattice of unreconstructed Ir(110) [encircled in magenta, with primitive reciprocal translations ${\vec{a}}_{Ir,1}^{*}$ and ${\vec{a}}_{Ir,2}^{*}$], the hexagonal Gr lattice [encircled in green, with primitive reciprocal translations ${\vec{a}}_{Gr,1}^{*}$ and ${\vec{a}}_{Gr,2}^{*}$], and the hexagonal ${\mathrm{MnBr}}_{2}$ lattice [encircled in orange, primitive reciprocal translations ${\vec{a}}_{MnBr_{2},1}^{*}$ and ${\vec{a}}_{MnBr_{2},2}^{*}$]. Gr and Ir(110) are epitaxially aligned with a Gr zigzag direction parallel to ${\left[001\right]}_{\mathrm{Ir}}$ [53]. ${\mathrm{MnBr}}_{2}$ forms a hexagonal 2D lattice epitaxially aligned to Gr, with the two hexagonal lattices rotated by 30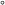 with respect to each other. However, the epitaxy is not perfect, as manifested by the azimuthal smearing of the ${\mathrm{MnBr}}_{2}$ diffraction spots and the presence of a faint, barely visible diffraction ring (highlighted by the segment of the dotted circle and the black arrow). The smearing of the diffraction spots becomes broader and the diffraction ring is more intense when the growth temperature is lower. As typical in van der Waals epitaxy, the lower the growth temperature, the larger the orientation scatter. Using the Ir diffraction spots as a reference, analyzing multiple LEED patterns yields a ${\mathrm{MnBr}}_{2}$ lattice parameter of 3.90  Å ± 0.01  Å. This value is in excellent agreement with both the experimental bulk ${\mathrm{MnBr}}_{2}$ lattice constant of 3.873  Å [54] and the theoretically predicted values for single‐layer ${\mathrm{MnBr}}_{2}$ [3, 21, 22]. Other spots not mentioned and visible in the LEED pattern can be traced back to the moiré of Gr with Ir(110) [53].

Figure 1b presents a scanning tunneling microscopy (STM) overview topograph of the same sample characterized by LEED, revealing two large first‐layer ${\mathrm{MnBr}}_{2}$ islands partially overlaid by smaller coalesced second‐layer islands on top. The islands exhibit a triangular morphology with mostly straight edges, consistent with the $C_{3v}$ symmetry expected for a single layer of ${\mathrm{MnBr}}_{2}$. At $V_{b}=-2.0$ V, the apparent height of the first‐layer island is measured to be 4.15 Å (see Figure S1). This value is lower than the expected geometric height of ${\mathrm{MnBr}}_{2}$ on Gr, since the bulk interlayer spacing of ${\mathrm{MnBr}}_{2}$ is 6.272 Å [17]. The underlying reason for this discrepancy is discussed in detail in the Section Apparent Height and Density of States. The contrast‐enhanced insets of the first‐layer islands in Figure 1b also display different stripe patterns. The stripe patterns are the moirés of single‐layer ${\mathrm{MnBr}}_{2}$ with the underlying substrate, which are discussed in more detail in the Section Super‐Moiré. The different orientations and appearances of the two stripe patterns result from the different orientations of the ${\mathrm{MnBr}}_{2}$ islands, consistent with the smeared LEED spots and the faint diffraction ring of ${\mathrm{MnBr}}_{2}$. The islands are rotated by $\approx 6^{\circ }$ with respect to each other.

Taking a closer look at Figure 1b, it is obvious that the first‐layer ${\mathrm{MnBr}}_{2}$ islands point in nearly opposite directions, where the triangular tip of the island in the upper part of the image points to the left and that in the lower part points to the right. The nearly opposite orientation of the large islands is consistent with the three‐fold symmetric ${\mathrm{MnBr}}_{2}$ growing on six‐fold symmetric Gr. The fact that the orientations are not exactly opposite is consistent with the azimuthally smeared ${\mathrm{MnBr}}_{2}$ reflections in the LEED pattern in Figure 1a.

Figure 1c shows an atomically resolved STM image of single‐layer ${\mathrm{MnBr}}_{2}$, which confirms a hexagonal lattice that is consistent with the LEED results. In addition to the atomic‐scale corrugation of ≈ 0.2 Å, the image exhibits nanoscale modulations. Most prominent are bright, nearly vertical stripes, which are the same as those visible in the contrast‐enhanced insets of Figure 1b. A small number of dark spots of atomic size are present in the atomically resolved image. Since their concentration does not show a dependence on growth or annealing temperature, they could result from a slight Br loss during the molecular sublimation in the Knudsen cell at 670 K and be Br vacancies.

The second‐layer islands in Figure 1b have two orientations with respect to the first‐layer islands. Either their triangular envelope aligns with their base or opposes it. From the analysis of many STM topographs, it is found that aligned and opposing envelopes of second‐layer islands are present in nearly equal proportions. As visualized in the ball model of Figure 1d, the aligned envelopes correspond to bulk ${\mathrm{MnBr}}_{2}$ which crystallizes in the ${\mathrm{CdI}}_{2}$ structure [17], also denoted 1T‐${\mathrm{MnBr}}_{2}$. In 1T‐${\mathrm{MnBr}}_{2}$, layers composed of octahedrally coordinated Mn ion planes sandwiched between Br ion planes are stacked exactly on top of each other. The envelopes of opposite orientations indicate a stacking fault as obvious from Figure 1e. Deviations from 1T stacking are well known for bulk ${\mathrm{MnBr}}_{2}$: above 623 K, ${\mathrm{MnBr}}_{2}$ in the ${\mathrm{CdCl}}_{2}$ structure is the stable phase, that is, the layers are rhombohedrally stacked, giving rise to a tripled c‐axis parameter [55]. This implies small free energy differences for variations in stacking, consistent with the formation of stacking faults under the non‐equilibrium conditions of MBE growth. In agreement with our observations, DFT calculations in the BiDB database [56] found identical energies for the two experimental ${\mathrm{MnBr}}_{2}$ stackings within the precision of DFT, that is, an energy difference of only 0.1 meV /Å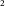.

### 2.2 Morphological Evolution with Temperature in Growth and Annealing

**FIGURE 2:**
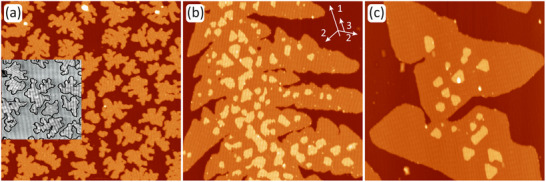
(a)–(c) STM topographs of ${\mathrm{MnBr}}_{2}$ on Gr/Ir(110) after deposition of ≈0.6 ML at 80 K, 300 K, and 400 K, respectively. The sample in (a) was briefly annealed to ≈150 K during transfer to the STM and then imaged at 77 K. Inset in (a) uses double gray scale to make the moiré on Gr/Ir(110) and the moiré on the ${\mathrm{MnBr}}_{2}$ islands simultaneously visible. Images in (b) and (c) were acquired at 300 K. The white arrows in (b) indicate the dendritic growth directions of the primary, secondary, and ternary dendrite branches. STM imaging parameters: (a) $V_{b}=-1$ V and $I_{t}=50$ pA, (b,c) $V_{b}=-2$ V and $I_{t}=50$ pA Image sizes: 160 nm × 160 nm.

Figure 2 compares the morphology of ${\mathrm{MnBr}}_{2}$ as a function of growth temperature. Figure 2a, taken at 77 K after growth at 80 K with unavoidable annealing to ≈150 K in between due to sample transfer, reveals fractal island growth with smooth edges and no preferential orientation. The smooth edges suggest limited edge diffusion during the annealing period. In the absence of annealing at ≈ 150 K, the islands would probably have retained an even more irregular fractal shape. As shown in the inset of Figure 2a, most of the islands exhibit a single well‐defined moiré orientation, except when grown together; an example of this is found in the upper left corner of the contrast‐enhanced inset. One island in the inset does not display a visible moiré, for which the reason will be discussed in Section Super‐moiré. The inset also shows the moiré of the Gr/Ir(110) substrate, which is always present, but much better visible for the specific bias used to take Figure 2a. The average moiré stripe periodicity is 3.3 nm [53], although due to the magnification effect of the Gr/Ir(110) moiré for strain and defects, substantial variations occur around this average. In any case, even when the ${\mathrm{MnBr}}_{2}$ moiré stripes are parallel to those of the substrate, their wavelength is substantially smaller. Second‐layer islands are largely absent. The average distance between islands of about 25 nm is much smaller than the typical separation between steps or other surface defects of about 150 nm. This implies the dominance of homogeneous nucleation.

Figure 2b, acquired at 300 K after growth at 300 K, shows a segment of a large dendritic island. While the main visible branch of the dendrite grows from bottom to top (direction labeled 1 in the inserted sketch in Figure 2b), secondary branches grow away from it (directions labeled 2), maintaining angles of approximately 120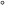. These angles correspond to the preferred growth directions, which are consistent with the three‐fold symmetry of the crystal structure. The island edges are smooth on a much larger length scale than in Figure 2a and displays undulations. Sharp grooves were observed between the secondary branches. Their origin can be traced back to the limited molecule supply from the diffusion field, which is preempted by the rapidly growing secondary branches and islands in the second layer. Some of the grooves are pinched, causing holes. The onset of tertiary branches is visible in Figure 2b (directions marked 3). From the dendritic appearance, the rather smooth edges and the simultaneous existence of grooves, it must be concluded that step edge diffusion is effective on length scales of the order 5 nm, but limited on large length scales of the order 50 nm. Searching the sample by moving the STM scan frame shows dendritic growth all over, but the substrate steps and defects give rise to heterogeneous nucleation, making a quantitative assessment of the nucleation density impossible to achieve. Qualitatively, the strong reduction in the island number density with increasing growth temperature is consistent with classical nucleation theory [57]. A large number of second‐layer islands nucleated on top of the dendritic island. This observation implies that the diffusion of ${\mathrm{MnBr}}_{2}$ molecules on the first‐layer ${\mathrm{MnBr}}_{2}$ islands is hampered compared to diffusion on Gr/Ir(110), likely due to a larger activation barrier for molecule migration. Second‐layer islands are seen to be reduced in number density next to the steps of the first‐layer islands. This implies that for molecules diffusing on a first‐layer island, the edge acts as a sink for molecules by incorporating them. Consequently, if there is any Ehrlich–Schwoebel barrier [57, 58, 59] for the descent of molecules from the first‐layer island, it must be very small.

Figure 2c taken at 300 K and grown at 400 K shows two first‐layer islands grown together. The right island is a good example of dendritic‐skeletal growth [57]. Its envelope is triangular, but its triangular sides are concave. The diffusion field has the strongest concentration gradient at the triangle tips; thus, the tips receive the largest supply of molecules. Although diffusion is efficient, transport along the edge is not fast enough to keep the edges straight, causing concave edges. Due to the higher growth temperature, the nucleation density of second‐layer ${\mathrm{MnBr}}_{2}$ islands is smaller compared to the 300 K case. The zone denuded of second‐layer islands adjacent to the first‐layer island edges has grown in width, consistent with enhanced molecule diffusion at higher temperatures. The second‐layer islands are compact and, if not coalesced, of hexagonal to triangular shape, since, due to their small size, diffusion is efficient in keeping the edges straight.

Taken together, the inferred picture of island nucleation and shape evolution is consistent with a strong increase with the growth temperature of (i) molecule diffusion on Gr/Ir(110) and (ii) step edge diffusion. The much higher diffusion coefficient of molecules on Gr/Ir(110) compared to that on the first‐layer ${\mathrm{MnBr}}_{2}$ islands implies that homogeneous nucleation has already ceased for the first‐layer islands at 300 K, while for the second‐layer islands, it remains homogeneous throughout the investigated temperature range.

Figure S2 for 400 K growth of ${\mathrm{MnBr}}_{2}$ on Gr/Ir(111) shows qualitatively the same behavior as for the 400 K growth on Gr/Ir(110). Nucleation, second‐layer nucleation, diffusion, and island shapes of ${\mathrm{MnBr}}_{2}$ appear to be similar on both substrates.

**FIGURE 3:**
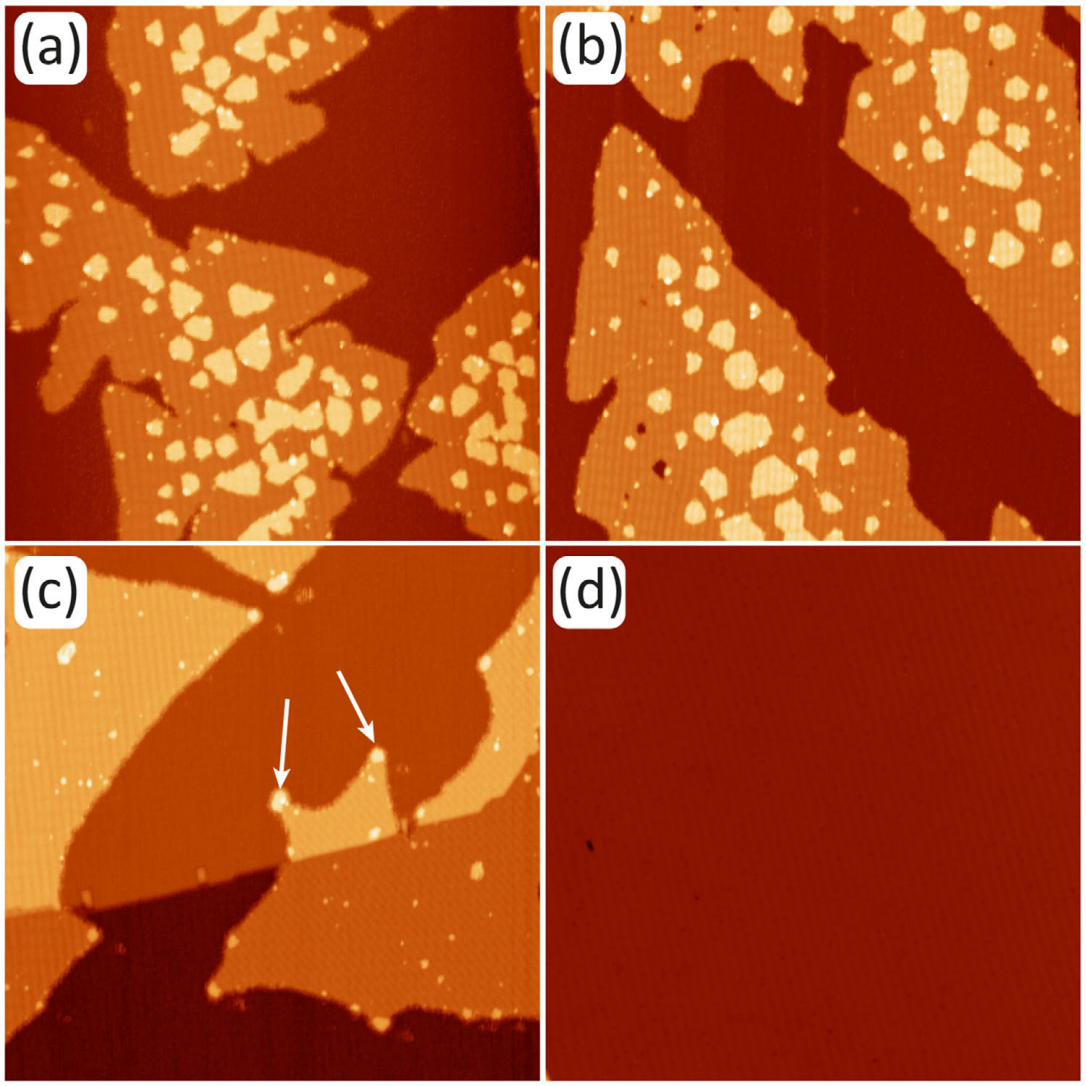
(a)‐(d) The ${\mathrm{MnBr}}_{2}$ sample of Figure 2(b) grown at 300 K is annealed in isochronal steps of 120 s successively to 370 K, 470K̇, 570 K, and 670 K, respectively. White arrows in (c) highlight two pinning centers. Imaging is conducted 300 K with $V_{b}$ = −2 V, $I_{t}=50$ pA, Image sizes: 160 nm × 160 nm.

Figure 3 presents an isochronal annealing sequence of the sample shown in Figure 2b grown at 300 K. The first two annealing steps to 370 (Figure 3a) and 470 K (Figure 3b) do not cause significant morphological changes. The island edges tend to become smoother, and at 470 K, the deep grooves of the dendrite branches disappear. Annealing to 570 K as represented by Figure 3c shows significant morphological changes. The second‐layer islands disappeared. First‐layer island steps show bright spots connected by concave‐step segments. These observations indicate the onset of ${\mathrm{MnBr}}_{2}$ molecule evaporation from the island edges. Due to their small radii of curvature, second‐layer islands have the highest vapor pressure and disappear first. The bright spots at the first‐layer island edges are pinning centers, presumably Mn clusters resulting from ${\mathrm{MnBr}}_{2}$ decomposition. The evaporation of ${\mathrm{MnBr}}_{2}$ molecules between the pinning centers causes these step segments to become concave, thereby lowering the vapor pressure at the edges. After annealing at 670 K (Figure 3d), no ${\mathrm{MnBr}}_{2}$ remains on the surface, indicating that complete evaporation to the gas phase has occurred. This result is consistent with the Knudsen cell temperature of 670 K used for the sublimation of ${\mathrm{MnBr}}_{2}$.

### 2.3 Apparent Height and Density of States

**FIGURE 4:**
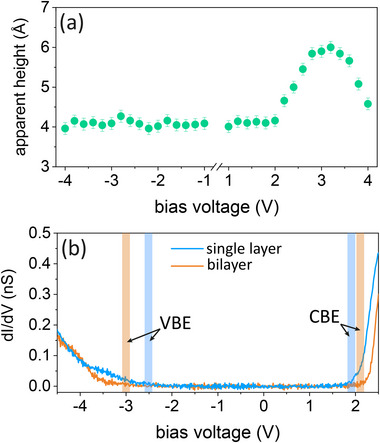
(a) Apparent height of single‐layer ${\mathrm{MnBr}}_{2}$ with respect to Gr for $I_{t}=20$ pA plotted as a function of $V_{b}$. (b) Constant‐height dI/dV spectra of single‐layer and bilayer ${\mathrm{MnBr}}_{2}$. The spectra are obtained with $V_{st}$ = 2.5 V, $I_{st}$ = 100 pA, $f_{mod}$ = 667 Hz, and $V_{mod}$ = 20 mV.

The apparent STM height of single‐layer ${\mathrm{MnBr}}_{2}$ on Gr/Ir(110) is plotted in Figure 4a as a function of the bias voltage $V_{b}$. Over a large range from –4 to 2 V it is rather insensitive to $V_{b}$ with an almost constant value of ∼4 Å. Only above $V_{b}=2\;V$ it increases, reaches at $V_{b}$ = 3.2 V a maximum of 5.95 ± 0.05 Å and then decreases again. The maximal measured height at $V_{b}$ = 3.2 V almost reaches the experimental c‐axis lattice parameter of bulk ${\mathrm{MnBr}}_{2}$, which is 6.272 Å [17]. We note that the precise apparent heights measured depend on $I_{t}$ and the tip state, but its $V_{b}$‐dependence, as represented in Figure 4a is generic.

Also for second‐layer islands, the apparent heights are in the range of 4 –6 Å, dependent on $V_{b}$, $I_{t}$, and the tip state. For an example height profile of a second‐layer island, see Figure S1. The situation is similar to that of ${\mathrm{CoCl}}_{2}$, where different apparent STM heights ranging from 4.8 to 8.0 Å and strong bias dependencies were reported [10, 11, 15]. Atomic force microscopy provides a height of 6.3 Å for ${\mathrm{CoCl}}_{2}$, reasonably close to the expected bulk value of 5.9 Å [60]. As discussed in the following, electronic effects are responsible for the variation in the apparent height measured by STM.

Figure 4b displays a differential conductance (dI/dV) spectrum acquired from a defect‐free region more than 10 nm from the edges of single‐layer ${\mathrm{MnBr}}_{2}$ (blue curve). The differential conductance is, in the first approximation, proportional to the local density of states of the sample. The valence band edge (VBE) is located at $V_{b}=\left(-2.5\pm 0.1\right)$ V and the conduction band edge (CBE) at $V_{b}=1.9\pm 0.1$ V. The resulting total gap of (4.4±0.2) eV makes ${\mathrm{MnBr}}_{2}$ an insulator.

Screening through the Gr substrate may be expected to have renormalized our measured bandgap to values smaller than of freestanding ${\mathrm{MnBr}}_{2}$, as was observed for instance, for ${\mathrm{MoSe}}_{2}$ [61] or ${\mathrm{MoS}}_{2}$ [62]. Consistent with this speculation, we observe a larger bandgap for bilayer ${\mathrm{MnBr}}_{2}$, for which screening through the Gr substrate is reduced. As shown in Figure 4b, for bilayer ${\mathrm{MnBr}}_{2}$ (red data) the VBE is located at $V_{b}=\left(-3.0\pm 0.1\right)$ V and the CBE at $V_{b}=\left(2.1\pm 0.1\right)$ V. The total resulting gap of (5.1±0.2) eV is significantly larger than the bandgap of the single‐layer. A similar trend has been reported for ${\mathrm{CoCl}}_{2}$ grown on graphite (see the Supporting Information of ref. [11]), where the bilayer displays a larger bandgap than the single‐layer. For freestanding single‐layer ${\mathrm{MnBr}}_{2}$ previous DFT calculations found a bandgap of 4.21[21] or 3.95 eV [22] and an even smaller bandgap for ${\mathrm{MnBr}}_{2}$ bulk. These results are consistent with ${\mathrm{MnBr}}_{2}$ being an insulator, but seem to underestimate the bandgap.

Evidently, the strong variation in apparent height, as shown in Figure 4a, is related to the electronic structure of ${\mathrm{MnBr}}_{2}$. In the range of the bandgap between $V_{b}=\left(-2.5\pm 0.1\right)$ V and $V_{b}=\left(1.9\pm 0.1\right)$ V tunneling is between the tip and the moiré substrate Gr/Ir(110). The tunnel barrier is composed of the vacuum barrier between the tip and ${\mathrm{MnBr}}_{2}$ and the barrier within ${\mathrm{MnBr}}_{2}$ between its two surfaces facing vacuum and Gr/Ir(110). The barrier within ${\mathrm{MnBr}}_{2}$ is defined by its CBE. Since the CBE is lower in energy than the vacuum level, the presence of ${\mathrm{MnBr}}_{2}$ enhances the tunneling probability and thus gives rise to a non‐zero height of ${\mathrm{MnBr}}_{2}$ in the constant current mode.

When $V_{b}$ decreases below −2.5 V, a new tunneling channel opens: electrons can then tunnel also out of the ${\mathrm{MnBr}}_{2}$ valence band. However, it is far below the Fermi level of the sample. Compared to electrons close to the sample Fermi level, their effective tunneling probability is lower by far, because of a much larger effective barrier height. Thus, the effect of tunneling out of the deep valence band states −2.5 eV below the Fermi level is neglectable compared to tunneling out of Gr/Ir(110) states near the Fermi level. Therefore, no significant change in the apparent barrier height is observed.

When $V_{b}$ increases above 1.9 V, tunneling into the ${\mathrm{MnBr}}_{2}$ conduction band sets in, thus giving rise to a new tunneling channel with a low effective barrier height. To maintain the same tunneling current under constant current tunneling conditions, this causes the tip to withdraw and gives rise to an increased apparent height. The decrease in apparent height beyond $V_{b}=3.2$ V indicates that above 3.2 eV the density of states in ${\mathrm{MnBr}}_{2}$ decreases again, as also evident in the DFT calculations of ref. [22].

### 2.4 Super‐Moiré

**FIGURE 5:**
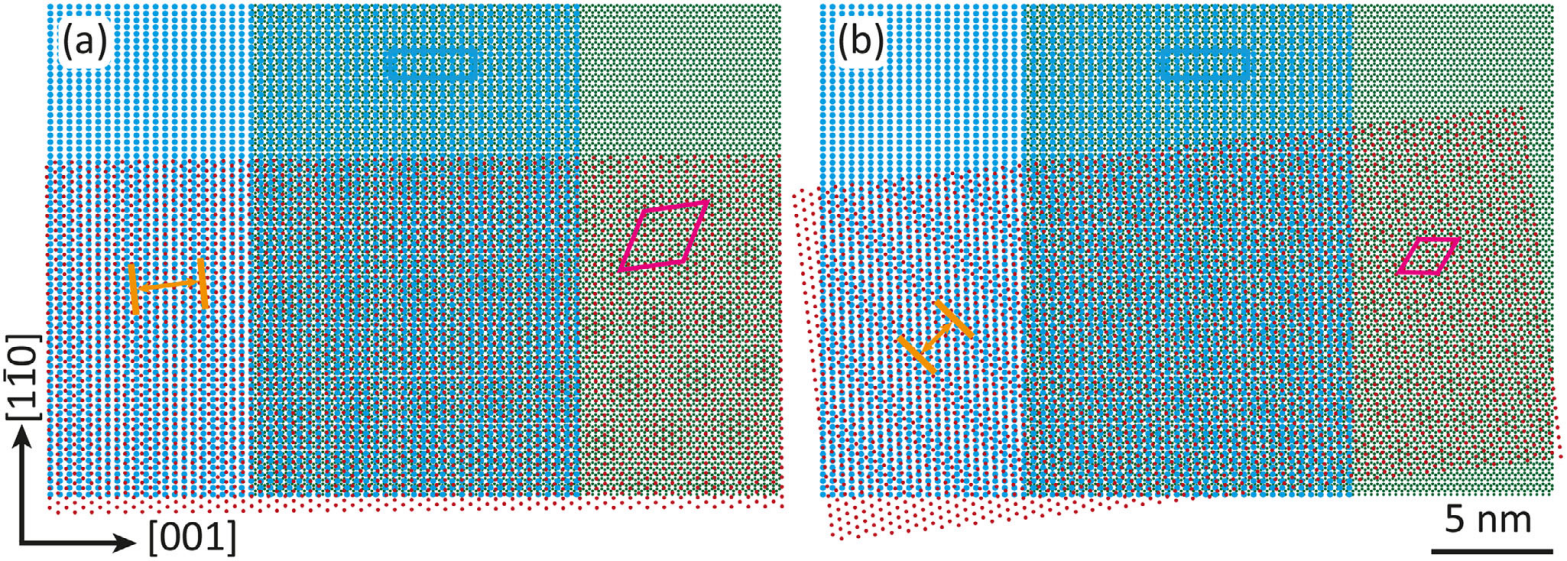
Ball model of moiré formation through overlay of the Ir(110) surface lattice (starting left), the Gr lattice (starting right), and one Br sublattice of ${\mathrm{MnBr}}_{2}$ (starting at the bottom). The ${\mathrm{MnBr}}_{2}$ lattices are rotated counterclockwise in (a) by $0.5^{\circ }$ and in (b) by $6.5^{\circ }$ with respect to the $\mathrm{Ir}[1\bar{1}0]$ direction. The moiré unit cells of Ir(110) with Gr and Gr with ${\mathrm{MnBr}}_{2}$ are indicated by blue rectangles and pink rhombuses, respectively. The wavelength of the moiré resulting from the interference between Ir(110) and ${\mathrm{MnBr}}_{2}$ is indicated by orange double arrows.

The first insight into the super‐moiré in the ${\mathrm{MnBr}}_{2}$/Gr/Ir(110) system is obtained by overlaying ball‐model representations of the three constituent lattices, as shown in Figure 5. The Ir(110) surface lattice (left) overlaps in the central area of both subfigures with the Gr honeycomb lattice (right). On these lattices, one Br sublattice (or equivalently the Mn sublattice) is overlaid (red balls, lattice shifted down) at two different angles. The angles of $0.5^{\circ }$ in Figure 5a and $6.5^{\circ }$ in Figure 5b for the dense‐packed rows of the ${\mathrm{MnBr}}_{2}$ lattice with respect to $\mathrm{Ir}[1\bar{1}0]$ were selected to reproduce qualitatively the moiré stripes of the two islands in Figure 1b. On the left of each subfigure, a clean stripe moiré formed by Ir(110) with ${\mathrm{MnBr}}_{2}$ is observed, in the upper middle the rectangular Gr/Ir(110) moiré (less pronounced), and on the right, a hexagonal moiré of Gr with ${\mathrm{MnBr}}_{2}$ evolves, as indicated by an orange double arrow, a blue rectangle, and a pink rhombus, respectively. In the lower middle, where the three lattices overlap, the ${\mathrm{MnBr}}_{2}$/Ir(110) moiré stripes survive, but become modulated due to the additional Gr lattice.

In fact, these stripes are the dominant features in wavelength and orientation in the moirés highlighted in Figure 1b. When the angle between the dense‐packed rows of the ${\mathrm{MnBr}}_{2}$ lattice with respect to $\mathrm{Ir}[1\bar{1}0]$ increases beyond $6.5^{\circ }$, the spacing of the stripes becomes smaller and reaches a minimum of 0.6 nm at $30^{\circ }$. Due to the low growth temperature, the islands in Figure 2a displays an almost random orientation and consequently a large scatter in the orientation and width of the moiré stripes. The absence of a visible stripe pattern in one of the fractal islands in the inset of Figure 2a is a consequence of a large angle near $30^{\circ }$, where the stripe pattern is not more resolved due to its small wavelength and the limited resolution on the scale of the topograph.

Although in the construction presented here, optically realized, it remains somewhat mysterious how a moiré of two lattices not in contact with each other, namely ${\mathrm{MnBr}}_{2}$ and Ir(110), can form the dominant STM corrugation feature in the ${\mathrm{MnBr}}_{2}$ islands.

**FIGURE 6:**
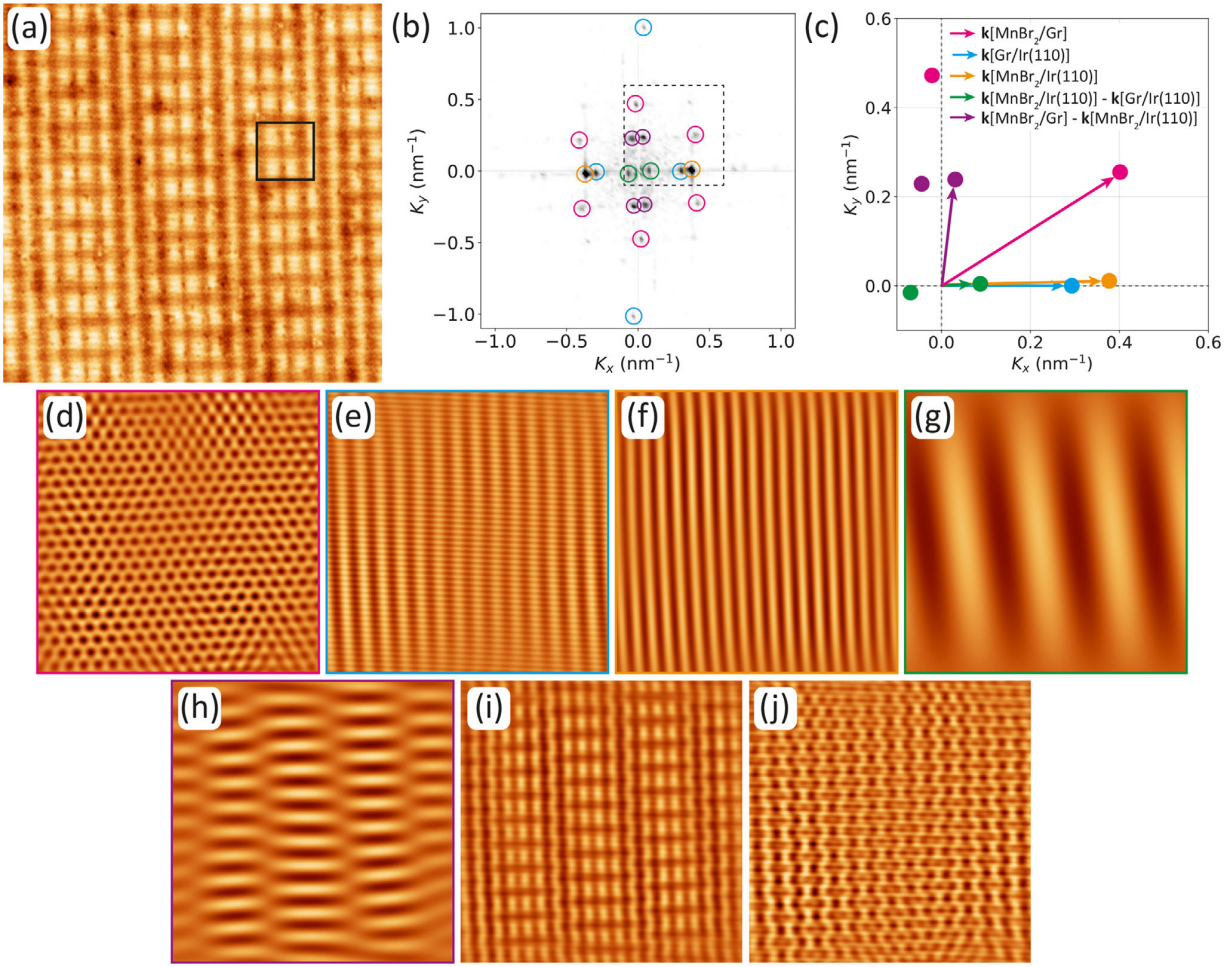
Fast Fourier transformation (FFT) analysis of the ${\mathrm{MnBr}}_{2}$/Gr/Ir(110) super‐moiré. (a) High resolution STM topography of ${\mathrm{MnBr}}_{2}$. Black box indicates size of atomic resolution topograph in Figure 1(b). (b) Inner part (small $\vec{k}$) of the FFT of the STM image shown in (a) with color‐coded circles surrounding the spots. (c) Enlarged schematics of dashed box in (b) using same color code. The relevant $\vec{k}$ vectors are indicated. (d)‐(h) are inverse FFTs using the spots in (b) encircled (d) pink, (e) blue, (f) orange, (g) green, and (h) purple. (i) is the inverse FFT using all spots encircled in (b), while (j) is the inverse FFT resulting from the spots encircled pink and blue. STM imaging parameters in (a) are $V_{b}=-1$ V and $I_{t}=50$ pA. Image sizes: 50 nm × 50 nm.

The high‐resolution topography shown in Figure 6a exhibits additional complex moiré features beyond what can be visualized in a ball model. With an angle of about $0.5^{\circ }$ between the dense‐packed ${\mathrm{MnBr}}_{2}$ rows and the $\mathrm{Ir}[1\bar{1}0]$ direction it corresponds to the situation visualized by the ball model in Figure 5a and highlighted in the upper inset of Figure 1b. To obtain a deeper understanding and to identify the Fourier components of the super‐moiré, we performed a fast Fourier transform (FFT) analysis of Figure 6a. The central part of the FFT limited to small $\vec{k}$ or large wavelengths is represented in Figure 6b, where the relevant peaks are encircled in color. An enlarged and schematic version of the dashed box in Figure 6b is shown in Figure 6c using the same color code. The inverse FFT analysis of sets of equally colored peaks provides insight into the main components of the super‐moiré.

When selecting only the FFT peaks encoded in pink, forming a hexagon in Figure 6b with wave vectors like $\vec{k}\left[{\mathrm{MnBr}}_{2}/\mathrm{Gr}\right]$, the inverse FFT results in Figure 6d. The image represents the hexagonal moiré between ${\mathrm{MnBr}}_{2}$ and Gr already visible in the ball model of Figure 5a where a unit cell of the moiré is indicated by a pink rhombus. It has a lattice parameter of 2.5 nm.

When selecting only the four FFT peaks encoded in blue in Figure 6b with wave vectors like $\vec{k}\left[\mathrm{Gr}/\mathrm{Ir}\left(110\right)\right]$, the resulting inverse FFT is shown in Figure 6e. The image represents the rectangular moiré between Ir(110) and Gr already visible in the ball model of Figure 5a where a unit cell of the moiré is indicated by a blue rectangle. The orthogonal periodicities of the Gr/Ir(110) moiré are 1.0 and 3.3 nm [53]. This moiré is also present between the ${\mathrm{MnBr}}_{2}$ islands in the contrast‐enhanced inset of Figure 2a, with the 3.3 nm well visible. The assignment is also supported by comparing the FFTs of plain Gr/Ir(110) and ${\mathrm{MnBr}}_{2}$/Gr/Ir(110) as done in Figure S3. Figure 6e also closely matches the topography of Gr/Ir(110) as shown in Figure S3a.

A traditional super‐moiré would result from differences or sums of the corresponding moiré wave vectors, i.e., from combinations like $\vec{k}\left[{\mathrm{MnBr}}_{2}/\mathrm{Gr}\right]-\vec{k}\left[\mathrm{Gr}/\mathrm{Ir}\left(110\right)\right]$. However, such Fourier components do not exist in the FFT. Instead, other components are present.

When selecting the two most intense FFT peaks encoded in orange with wave vectors $\pm \vec{k}\left[{\mathrm{MnBr}}_{2}/\mathrm{Ir}\left(110\right)\right]$, the resulting inverse FFT is shown in Figure 6f. The image represents the interference between the ${\mathrm{MnBr}}_{2}$ and Ir(110) lattices. The pattern has a row spacing of 2.7 nm – distinctly smaller than the 3.3 nm periodicity of Gr/Ir(110) – and gives rise to a pronounced line pattern in the ${\mathrm{MnBr}}_{2}$/Gr/Ir(110) super‐moiré. This line pattern is also clearly visible in Figure 5a on the left, where the Ir(110) surface rows overlap with the ${\mathrm{MnBr}}_{2}$ lattice. Its periodicity is indicated in Figure 5a by an orange double arrow. As mentioned above, this virtual moiré of two lattices not in contact with each other dominates the STM corrugation in the ${\mathrm{MnBr}}_{2}$ islands and gives rise to the stripes visible still in larger‐scale topographs of ${\mathrm{MnBr}}_{2}$ islands, that is, to the stripes on ${\mathrm{MnBr}}_{2}$ islands highlighted in the insets of Figure 1b and 2a.

When selecting only the FFT peaks encoded in green, the resulting inverse FFT is shown in Figure 6g. These spots are due to the difference in the wave vector of the orange and blue spots, that is, from combinations like $\vec{k}\left[{\mathrm{MnBr}}_{2}/\mathrm{Ir}\left(110\right)\right]-\vec{k}\left[\mathrm{Gr}/\mathrm{Ir}\left(110\right)\right]$ in Figure 6b,c. The superposition of the two corresponding stripe patterns results in a beating pattern with a period of approximately 13.5 nm. This modulation is visible in the STM topograph of Figure 6a.

Finally, when selecting only the FFT peaks encoded in purple, the resulting inverse FFT is shown in Figure 6h. These spots are due to the difference in the wave vectors of the pink and orange spots, that is, from combinations like $\vec{k}\left[{\mathrm{MnBr}}_{2}/\mathrm{Gr}\right]-\vec{k}[{\mathrm{MnBr}}_{2}/\mathrm{Ir}\left(110\right]$.

When selecting all FFT peaks encircled in Figure 6b, the resulting FFT is shown in Figure 6(i). It reproduces all relevant features of the complex super‐moiré, as obvious from a comparison with the STM topograph of Figure 6a. In order to reproduce the super‐moiré, the ${\mathrm{MnBr}}_{2}$/Ir(110) moiré is indispensable. The related wave vectors, like $\vec{k}[{\mathrm{MnBr}}_{2}/\mathrm{Ir}\left(110\right]$, are contained in the orange, green, and purple spots. Selecting only the spots related to the ${\mathrm{MnBr}}_{2}$/Gr and Gr/Ir(110) moirés (spots encircled pink and blue) does not provide an adequate description of the super‐moiré as visible from Figure 6j.

**FIGURE 7:**
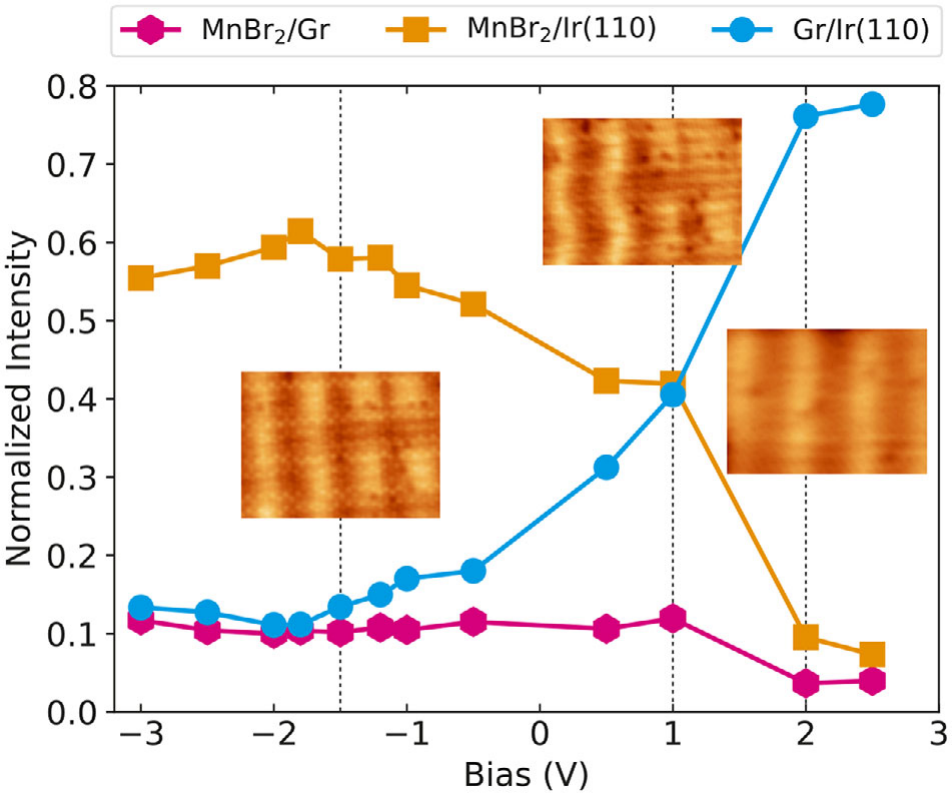
Bias voltage dependence of the six pink, four blue, and two orange Fourier peaks of Figure 6(b) as color‐coded in Figures 6(b) and (c). These peaks are linked to wave vectors like $\vec{k}\left[{\mathrm{MnBr}}_{2}/\mathrm{Gr}\right]$, $\vec{k}\left[{\mathrm{MnBr}}_{2}/\mathrm{Gr}\right]$, and $\vec{k}\left[{\mathrm{MnBr}}_{2}/\mathrm{Ir}\left(110\right)\right]$, respectively. The intensity of each component is normalized with the sum of all FFT peaks encircled in Figure 6(b). STM topographs in insets are 11 nm × 8 nm and taken at $V_{b}=-1.5$ V, +1 V, and +2 V as indicated by arrows and with $I_{t}=50$ pA.

Additional insight into the origin of the super‐moiré of ${\mathrm{MnBr}}_{2}$ on Gr/Ir(110) is obtained from the analysis of the bias dependence of its Fourier components. Figure 7 plots the relative FFT intensities of the pink, orange and blue peaks of Figure 6b linked to the ${\mathrm{MnBr}}_{2}$/Gr, ${\mathrm{MnBr}}_{2}$/Ir(110) and Gr/Ir(110) moirés, respectively. The pink and orange components are stable for $V_{b}\leq +1$ V, with the orange component by far most intense. At $V_{b}=+2$ V, the components drop close to zero. The blue component is for negative $V_{b}$ on a low and quite stable level, increases at positive $V_{b}$ and jumps to a new plateau for $V_{b}\geq +2$ V, where it is the only component with significant intensity. The increase of the wavelength from 2.7 nm (related to $\vec{k}\left[{\mathrm{MnBr}}_{2}/\mathrm{Ir}\left(110\right)\right]$) to 3.3 nm (related to $\vec{k}\left[\mathrm{Gr}/\mathrm{Ir}\left(110\right)\right]$) with increasing bias voltage is visualized by the STM insets in Figure 7.

The interpretation of these observations is straightforward. At positive bias with of $V_{b}\geq +2$ V tunneling is into the conduction band of ${\mathrm{MnBr}}_{2}$. Thereby, the ${\mathrm{MnBr}}_{2}$/Gr interface as well as the virtual ${\mathrm{MnBr}}_{2}$/Ir(110) interface are invisible to the tunneling current. The experimental corrugation is due to the height variation of the Gr/Ir(110) substrate, which is imposed on the soft ${\mathrm{MnBr}}_{2}$ layer. The geometrically imprinted Gr/Ir(110) moiré leaves the related blue Fourier component as dominant, while all other components vanish.

For $V_{b}\leq 1.9$ V tunneling is in the ${\mathrm{MnBr}}_{2}$ bandgap, that is, tunneling is *through* ${\mathrm{MnBr}}_{2}$ directly to the ${\mathrm{MnBr}}_{2}$/Gr interface. This explains why the interface‐related components are much larger than when tunneling into the conduction band. The tunneling current in this bias range is dominated by states around the Fermi level. Specifically, at all negative bias voltages electrons at the Fermi level have the lowest tunneling barrier and thus dominate the tunneling current. This explains the relative constancy of the Fourier components in this bias range. As noted above, when discussing the apparent height of single‐layer ${\mathrm{MnBr}}_{2}$, even when at $V_{b}\leq -2.5$ V, tunneling out of the valence band becomes possible, this tunneling channel is insignificant due its large tunneling barrier.

**FIGURE 8:**
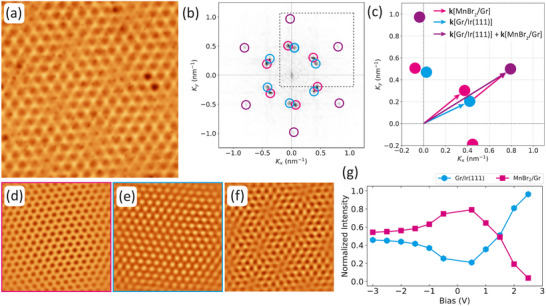
FFT analysis of the ${\mathrm{MnBr}}_{2}$/Gr/Ir(111) super‐moiré. (a) Moiré resolution STM topography of ${\mathrm{MnBr}}_{2}$. (b) Inner part (small $\vec{k}$) of the FFT of the STM image shown in (a) with color‐coded circles surrounding the spots. (c) Schematic enlarged section of dashed box in (b) using same color code. The relevant $\vec{k}$ vectors are indicated. (d) and (e) are inverse FFTs using the spots in (b) encircled (d) pink and (e) blue, respectively. (f) is the inverse FFT using all spots encircled in (b) pink and blue. (g) Bias voltage dependence of the six pink and six blue Fourier peaks of (b). These peaks are linked to wave vectors like $\vec{k}\left[{\mathrm{MnBr}}_{2}/\mathrm{Gr}\right]$ and $\vec{k}\left[\mathrm{Gr}/\mathrm{Ir}\left(111\right)\right]$, respectively. The intensity of each component is normalized with the sum of all FFT peaks encircled (b). STM imaging parameters in (a) are $V_{b}$ = –2 V, 100 pA. Image size is 35 nm × 35 nm.

The uniqueness of the ${\mathrm{MnBr}}_{2}$/Gr/Ir(110) super‐moiré is illuminated by comparing it to another three‐lattice system forming a super‐moiré. Figure 8 investigates the case of ${\mathrm{MnBr}}_{2}$/Gr/Ir(111), of which Figure 8a shows an STM topograph taken at the same $V_{b}$ as Figure 6a. It is dominated by the moiré of ${\mathrm{MnBr}}_{2}$ with Gr (pink encircled components in Figure 8b with inverse FFT in Figure 8d). The hexagonal Gr/Ir(111) moiré [66] with its 2.53 nm periodicity and 0.4 Å corrugation is also present in the FFT, giving rise to the blue encircled components in Figure 8b with inverse FFT in Figure 8e. The two moirés are slightly tilted with respect to each other. The super‐moiré results from their interference. In the inverse FFT shown in Figure 8f of the spots corresponding to the two moirés (encircled blue and pink in Figure 8b), a hexagonal super‐moiré with a periodicity of ≈13.2 nm becomes obvious, as is in the original STM topograph of Figure 8a . As there are only a few super‐moiré unit cells present in the original STM topograph, the difference wave vectors $\vec{k}\left[{\mathrm{MnBr}}_{2}/\mathrm{Gr}\right]-\vec{k}\left[\mathrm{Gr}/\mathrm{Ir}\left(111\right)\right]$ are presumably too low in intensity to be visible in the FFT. From the analysis of Figure 8 we conclude: (i) The FFT of the super‐moiré shown in Figure 8b displays no virtual ${\mathrm{MnBr}}_{2}$/Ir(111) component. (ii) The super‐moiré is established by creating an inverse FFT only from the spots related to the moirés of ${\mathrm{MnBr}}_{2}$/Gr and Gr/Ir(111), as shown in Figure 8f. This is the expected way of super‐moiré formation, but unlike the case of ${\mathrm{MnBr}}_{2}$/Gr/Ir(110), where the corresponding inverse FFT in Figure 6j does not reproduce the super‐moiré. (iii) The bias dependence of the interface Fourier component ${\mathrm{MnBr}}_{2}$/Gr and of the geometric Fourier component Gr/Ir(111) shows a very similar behavior as the corresponding components of ${\mathrm{MnBr}}_{2}$/Gr/Ir(110): below the CBE the interface component dominates the Fourier intensity, while in the conduction band only the geometric component is relevant (compare Figure 8g).

The unique feature of the ${\mathrm{MnBr}}_{2}$/Gr/Ir(110) is thus the existence and relevance of the virtual ${\mathrm{MnBr}}_{2}$/Ir(110) moiré, that is, of the ${\mathrm{MnBr}}_{2}$ and Ir(110) lattices that are not in contact with each other. Insight into the origin of this unique feature is provided by comparing Gr/Ir(110) with Gr/Ir(111).

Gr is physisorbed to Ir(111) with only a slight chemical modulation [63]. The C‐Ir distance is for all C atoms larger than 3.2 Å, thus of a typical van der Waals distance [63, 64]. There is no charge redistribution in or transfer to/from Gr that has the periodicity of the underlying Ir(111) surface lattice (see Figure 2 in ref. [63]). The Gr Dirac cone remains largely intact [65]. The Ir(111) lattice cannot be detected by STM through the Gr cover.

**FIGURE 9:**
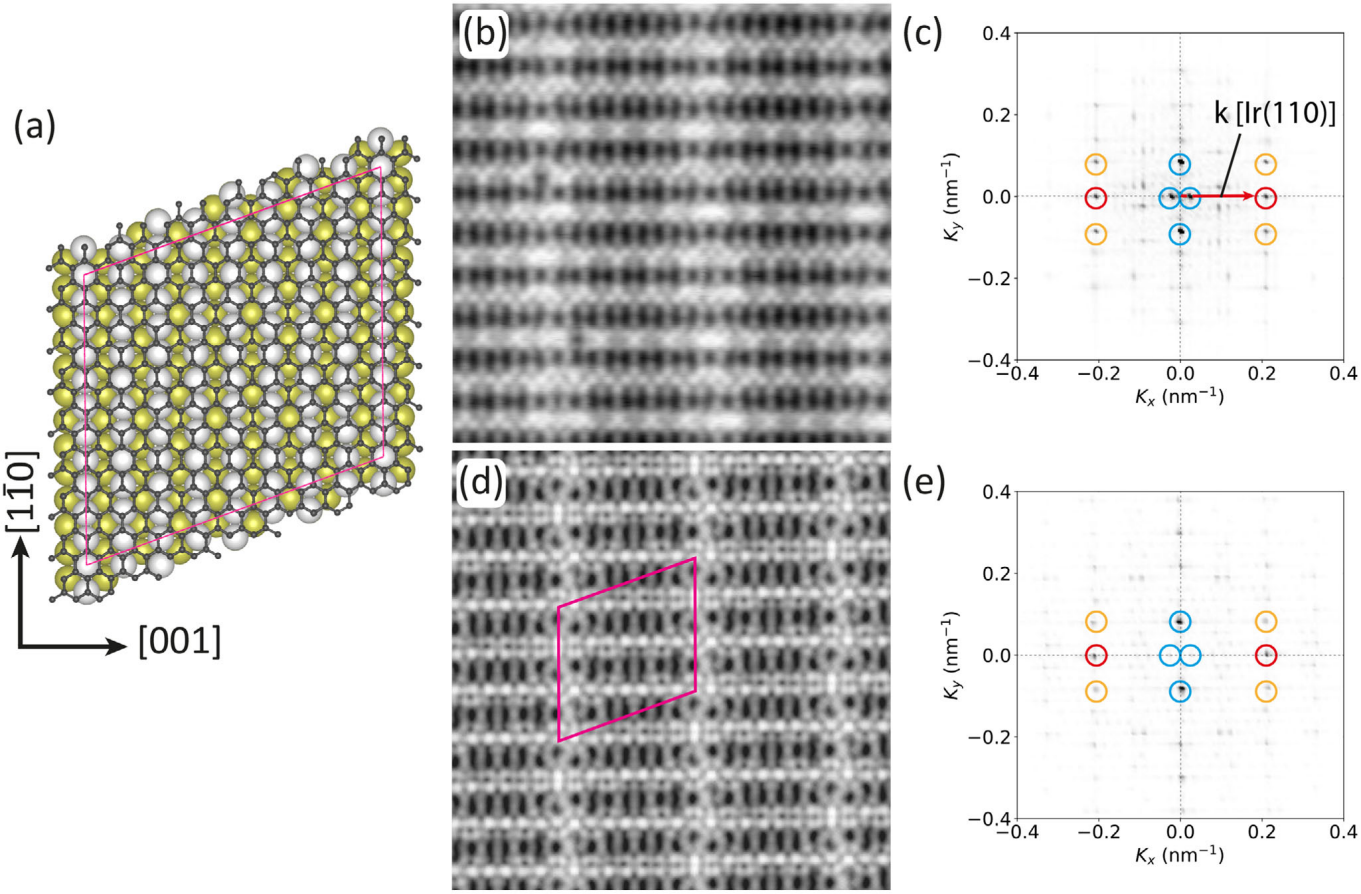
Ir(110) rows visible through Gr on Ir(110). (a) Ball model of Gr on Ir(110). Green gold balls: bottom‐layer Ir atoms; white balls: top‐layer Ir atoms; dark gray balls: C atoms. A pink rhombus indicates the supercell size in the DFT calculations. (b) STM topograph of Gr/Ir(110) moiré in high resolution. Image size: 10 nm × 10 nm. $V_{b}=-0.1$ V and $I_{t}=1$ nA. (c) FFT of (b). Moiré spots are encircled blue, spots due to Ir(110) dense‐packed rows are encircled red, spots that are $\vec{k}$‐vector sum of blue and red spots are encircled orange. (d) Simulated STM image for $V_{b}=-0.1$ V plotting the partial charge density in the energy interval $eV_{b}=\left[-0.1\mathrm{eV};0\right]$ above the surface for an isosurface value of $4.2\times 10^{-11}$ e/Å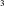. (e) FFT of (d).

Gr is chemisorbed to Ir(110) with a pronounced modulation between strong and weak chemisorption [53]. The C atoms above the dense‐packed rows of the Ir(110) top layer (light beige in Figure 9a) are strongly chemisorbed (distances of C‐Ir down to 2.1 Å), while C atoms sitting in between the densely packed rows (above the furrows, yellow in Figure 9a) are weakly chemisorbed (distances C‐Ir up to 3.1 Å). There is a strong charge redistribution and transfer of charge to Gr near the Fermi level with a periodicity of the underlying Ir(110) lattice (see Figures 3 and 4 of ref. [53]). The Dirac cone is destroyed for Gr on Ir(110) with a substantial density of states, where it might be expected.

Figure 9b is a high‐resolution STM topograph of the Gr/Ir(110) moiré with $V_{b}=-0.1$ V. In addition to the moiré itself, the periodicity of the dense‐packed top layer rows of Ir(110) with their spacing of 3.84 Å are well visible in the topograph. In the corresponding FFT of Figure 9c the spots related to the Ir rows encircled in red are the most intense spots except of those of the moiré itself. Note also that linear combinations of the moiré spots $\vec{k}[\mathrm{Gr}/\mathrm{Ir}\left(110\right]$ and the row spots with $\vec{k}\left[\mathrm{Ir}\left(110\right)\right]$ are intense (encircled orange). This implies that at the interface, the bromine lattice of ${\mathrm{MnBr}}_{2}$ with vectors $\vec{k}\left[{\mathrm{MnBr}}_{2}\right]$ is directly superimposed with the Ir(110) row spacing to form the virtual ${\mathrm{MnBr}}_{2}$/Ir(110) moiré with wave vectors $\vec{k}[{\mathrm{MnBr}}_{2}/\mathrm{Ir}\left(110\right]$. The presence of spots linked to the Ir(110) rows with $\vec{k}\left[\mathrm{Ir}\left(110\right)\right]$ and linear combinations of this $\vec{k}$‐vector with those of the moiré are generic and observed for a large range of tunneling parameters.

As example, for a tunneling resistance smaller by a factor of 20 compared to Figure 9b also the Gr lattice is resolved (see Figure 1c of ref. [53]). Nevertheless, the corresponding FFT shown in Figure S2 of ref. [53] still displays high intensity for the Ir(110) surface lattice.

Our inferences are supported by DFT calculations. Figure 9d shows the corresponding simulated STM topograph of Gr/Ir(110), which displays a pattern strikingly similar to the experimental STM topograph. In addition to the moiré pattern, the periodicity of the Ir(110) rows dominates the simulated STM topograph. This is underlined by the high intensity of the component encircled red in the corresponding FFT of Figure 9e. Also in a contour plot of the total charge density the Ir(110) row periodicity is clearly present as shown Figure S4. In conclusion, the probability density of the wave functions of Gr/Ir(110) extending into the vacuum, where ${\mathrm{MnBr}}_{2}$ is to be placed, carries a strong Fourier component representing the Ir(110) row periodicity.

Additional DFT calculations were performed to obtain information on the interaction between the ${\mathrm{MnBr}}_{2}$ and the Gr/Ir(110) substrate. Forced by computational limitations, we designed a smaller supercell that still correctly describes the chemistry at the ${\mathrm{MnBr}}_{2}$ and Gr/Ir(110) interface (see Figure S5 and Note S5). Overall, the ${\mathrm{MnBr}}_{2}$ is weakly physisorbed to Gr/Ir(110) with an adsorption energy of 14 meV/Å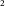. No hybridization of ${\mathrm{MnBr}}_{2}$ with its substrate is identified, but a small charge transfer from the substrate to the contact Br atoms is found. It is therefore plausible that a lateral modulation of the charge transfer is of relevance for the formation of the virtual ${\mathrm{MnBr}}_{2}$/Ir(110) moiré.

The imprint of the Ir(110) row periodicity on the Gr/Ir(110) moiré and the formation of the virtual ${\mathrm{MnBr}}_{2}$/Ir(110) moiré are unrelated to the well documented transparency of Gr on weakly interacting noble metal substrates. On Au(111) [66], Ag(111) [66, 67], and Cu(111) [68] tunneling conditions could be tuned to detect either quasiparticle scattering of metal surface state electrons or Gr and its moiré. The apparent transparency of Gr was explained by the slower decay of the surface state compared to the Gr local density of states into the vacuum [68].

However, in the present case, Gr on Ir(110) is strongly interacting with the substrate, rather than weakly. This is already evident from the fact that the Gr cover lifts the nanofacet reconstruction of Ir(110) [69]. ARPES and DFT calculations prove the absence of a Dirac cone [53] and thus a substantial inhomogeneity of the binding of C atoms to the substrate. The imprint of the spacing of the rows of the Ir(110) top layer on Gr is due to the fact that C atoms above the Ir(110) rows are strongly hybridized with the substrate, while those above the furrows interact much weaker with Gr. The chemical inhomogeneity of the C atoms in Gr/Ir(110) was already used to pattern adsorption [53]. It is this inhomogeneity, that enters that enters the formation of the virtual ${\mathrm{MnBr}}_{2}$/Ir(110) moiré and the unconventional structure of the ${\mathrm{MnBr}}_{2}$/Gr/Ir(110) super‐moiré.

## 3 Conclusion

At 400 K growth, ${\mathrm{MnBr}}_{2}$ is nearly epitaxially aligned to Gr with densely packed rows rotated by 30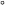, while at lower temperatures the orientation of ${\mathrm{MnBr}}_{2}$ islands scatters more around the preferred epitaxial relation, as typical in van der Waals epitaxy. ${\mathrm{MnBr}}_{2}$ exhibits a hexagonal lattice with a lattice parameter of 3.90 ± 0.01 Å, consistent with bulk 1T‐${\mathrm{MnBr}}_{2}$. The ${\mathrm{MnBr}}_{2}$ island morphology evolves from fractal to compact dendritic‐skeletal islands as temperature increases. Nucleation is homogeneous at low temperatures but becomes heterogeneous at defects and step edges at room temperature and above. Second‐layer nucleation is more probable than on bare Gr, while step‐edge barriers are insignificant. Upon annealing, sublimation begins at 570 K and ${\mathrm{MnBr}}_{2}$ is fully desorbed at 670 K. A comparative growth experiment suggests for single‐layer ${\mathrm{MnBr}}_{2}$ a similar growth behavior and structure on Gr/Ir(111). STS reveals a bandgap for single‐layer ${\mathrm{MnBr}}_{2}$ of approximately 4.4 eV with the valence band at ≈ –2.5 eV and conduction band at ≈ +1.9 eV. When tunneling into the ${\mathrm{MnBr}}_{2}$ conduction band, the apparent STM height reaches 5.95 Å, almost the bulk interlayer spacing, while in the bandgap it remains rather constant at the low value of 4 Å.

A complex super‐moiré arises from the interplay of ${\mathrm{MnBr}}_{2}$, Gr, and Ir(110). Its strongest Fourier component corresponds to a virtual moiré between ${\mathrm{MnBr}}_{2}$ and Ir(110), although the two lattices are not in contact. In real space, this virtual moiré causes a dominant 2.7 nm stripe pattern. Moreover, the linear combinations of these moiré wave vectors with the ones of the Gr/Ir(110) moiré with a 3.3 nm stripe periodicity give rise to a super‐moiré component with a 13.5 nm beating pattern. The same virtual ${\mathrm{MnBr}}_{2}$/Ir(110) moiré also enters the second super‐moiré component due to linear combinations of its wave vectors with those of the ${\mathrm{MnBr}}_{2}$/Gr moiré, which gives rise to orthogonal stripe segments in real space.

The analysis of the bias dependence of the Fourier components makes plain that the super‐moiré is present only in the bandgap of ${\mathrm{MnBr}}_{2}$ when tunneling is from or to the ${\mathrm{MnBr}}_{2}$/Gr interface, while when tunneling is into the conduction band, the corrugation of ${\mathrm{MnBr}}_{2}$ just reflects the corrugation of the Gr/Ir(110) moiré. Comparative experiments for ${\mathrm{MnBr}}_{2}$ on Gr/Ir(111) display the same behavior of a geometric moiré when tunneling into the conduction band and an interface super‐moiré present in the bandgap. However, a virtual moiré is present only in the ${\mathrm{MnBr}}_{2}$/Gr/Ir(110) case. Its existence is traced back to the inhomogeneous chemical binding of Gr to Ir(110), which causes an electronic and chemical modulation of Gr with 3.84 Å periodicity of the dense‐packed Ir(110) top layer rows, and thus also a modulation in the interaction with ${\mathrm{MnBr}}_{2}$.

Overall, this comprehensive characterization of an example 2D TMDH will be useful for understanding the growth, structure, and super‐moirés of other 2D materials in this class. The first characterization of a super‐moiré composed of lattices with different symmetries, broadened our understanding of how super‐moirés can emerge. In addition, virtual moirés of lattices that are not in contact with each other may be of primary relevance in the formation of super‐moirés. Since single‐layer ${\mathrm{MnBr}}_{2}$ is expected to host magnetic order and polarons, it will be interesting to explore whether any of these are affected by the super‐moiré of ${\mathrm{MnBr}}_{2}$/Gr/Ir(110).

## 4 Experimental Section

The experiments were carried out in two ultrahigh vacuum systems with base pressures below $1\times 10^{-10}$ mbar. Each system was equipped with standard MBE growth facilities, LEED or microchannel plate LEED (MCP‐LEED), and STM operated at temperatures of 300 K, 77 K, and 1.7 K.

Ir(110) was cleaned and prepared in its unreconstructed state by cycles of 4.5 keV ${\mathrm{Xe}}^{+}$ ion sputtering, flash annealing to 1510 K, and subsequent cooling to 400 K in $1\times 10^{-7}$ mbar oxygen pressure. Ir(111) was cleaned by cycles of 1 keV ${\mathrm{Ar}}^{+}$ sputtering and subsequent flash annealing to 1510 K.

Single‐crystal Gr was grown by heating cleaned unreconstructed Ir(110) to 1510 K and exposing it to $3\times 10^{-7}$ mbar ethylene for 240 s [53]. On Ir(111), Gr was grown by exposing the clean Ir(111) to $1\times 10^{-7}$ mbar ethylene at 300 K for 120 s, flash annealing to 1470 K without ethylene, and again exposure to $3\times 10^{-7}$ mbar ethylene for 600 s at 1370 K [70]. The quality of the as‐grown Gr/Ir(110) or Gr/Ir(111) was checked by LEED and STM.

${\mathrm{MnBr}}_{2}$ was grown by sublimation of ${\mathrm{MnBr}}_{2}$ molecules from ${\mathrm{MnBr}}_{2}$ powder in a Knudsen cell heated to 670 K. The Knudsen cell was placed 8 cm from the sample, producing a deposition rate of $3\times 10^{-4}$ ML/s. Here, a monolayer (ML) corresponds to complete surface coverage by a single layer of ${\mathrm{MnBr}}_{2}$, equivalent to $7.6\times 10^{18}$ ${\mathrm{MnBr}}_{2}$ molecules $m^{-2}\;s^{-1}$.

LEED patterns were acquired at 300 K using electron energies ranging from 90 to 150 eV. For MCP‐LEED measurements, distortions in the reciprocal space due to the flat microchannel plate geometry were corrected using a Python script.

STM was performed at 300 K in a variable temperature, and at 77 and 1.7 K in a bath cryostat STM system. Constant‐current topographies were recorded with sample bias $V_{b}$ and tunneling current $I_{t}$, as detailed in the respective figure captions. The STM images were analyzed and processed (plane subtraction, contrast correction) using WSxM software [71]. STS spectra were obtained at 1.7 K with stabilization bias $V_{st}$ and stabilization current $I_{st}$ using the standard lock‐in technique with modulation frequency $f_{mod}$ and modulation voltage $V_{mod}$, as specified in the captions.

Our ab initio density functional theory (DFT) [72, 73] calculations were carried out using the projector augmented wave (PAW) method [74] as implemented in the Vienna Ab initio Simulation Package (VASP) [75, 76, 77]. To account for the van der Waals interactions in the Gr/Ir(110) and ${\mathrm{MnBr}}_{2}$/Gr/Ir(110) systems, we employed the non‐local vdW‐DF2 correlation energy functional [78] combined with a re‐optimized B86b exchange functional [79, 80]. Gr on Ir(110) was modeled using a slab comprising three Ir layers and an in‐plane unit cell of ≈32.86×30.12 Å, amounting to 614 atoms (350 C and 264 Ir). The ${\mathrm{MnBr}}_{2}$/Gr/Ir(110) system was simulated by using an in‐plane unit cell of ≈7.04×28.46 Å that contains 192 atoms (i.e., 14 Mn, 28 Br, 84 C and 66 Ir). The ground‐state geometry and electronic structure were determined using a plane‐wave kinetic energy cutoff of 500 eV and a threshold for Hellmann–Feynman forces of ≈0.005 eV/Å.

## Conflicts of Interest

The authors declare no conflict of interest.

## Supporting information




**Supporting File**: smll72222‐sup‐0001‐SuppMat.pdf

## Data Availability

The data that support the findings of this study are available from the corresponding author upon request.
