# Supplementary material for: MnBr2 on the Graphene on Ir(110) Substrate: Growth, Structure, and Super‐Moiré
Source: Small. 2026 Jan 5;22(11):e09471. doi: 10.1002/smll.202509471 (PMC12921553; doi:10.1002/smll.202509471)
Supplement: Supplementary file 1 — Supporting File: smll72222‐sup‐0001‐SuppMat.pdf [file SMLL-22-e09471-s001.pdf]

Supplementary Information: $MnBr_2$ on the graphene on Ir(110) substrate: growth, structure, and super-moiré

Affan Safeer,[1,*] Oktay Güleryüz,[1] Nicolae Atodiresei,[2]
Wouter Jolie,[1] Thomas Michely,[1] and Jeison Fischer[1]

[1]*II. Physikalisches Institut, Universität zu Köln, Zülpicher Straße 77, 50937 Köln, Germany*

[2]*Peter Grünberg Institut (PG-1), Forschungszentrum Jülich, Wilhelm-Johnen-Straße, D-52428 Jülich, Germany*

* safeer@ph2.uni-koeln.de

CONTENTS

Supplementary Note 1. Apparent height of $MnBr_2$ 3

Supplementary Note 2. Comparison of single layer $MnBr_2$ grown on Gr/Ir(111) and Gr/Ir(110) 4

Supplementary Note 3. Comparison of Gr/Ir(110) with $MnBr_2$/Gr/Ir(110) 5

Supplementary Note 4. Contour of constant total charge density above Gr/Ir(110) 6

Supplementary Note 5. DFT simulation of $MnBr_2$/Gr/Ir(110) 7

References 8

Supplementary Note 1. Apparent height of $MnBr_2$

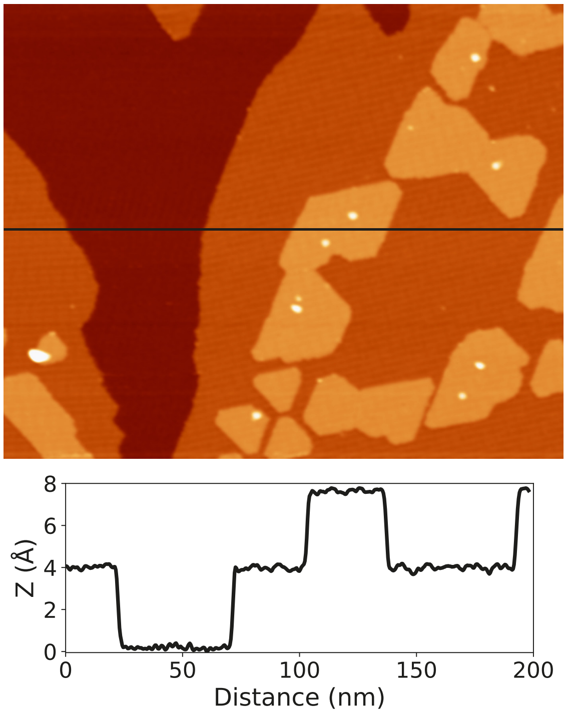

Figure S1. STM topograph of $MnBr_2$ on Gr/Ir(110). Lower panel shows the height profile along the black line in the STM topograph. STM image is obtained at 300 K with (b) V_b = -2 V, 50 pA. Image size: 200 nm × 160 nm

Supplementary Note 2. Comparison of single layer $MnBr_2$ grown on Gr/Ir(111) and Gr/Ir(110)

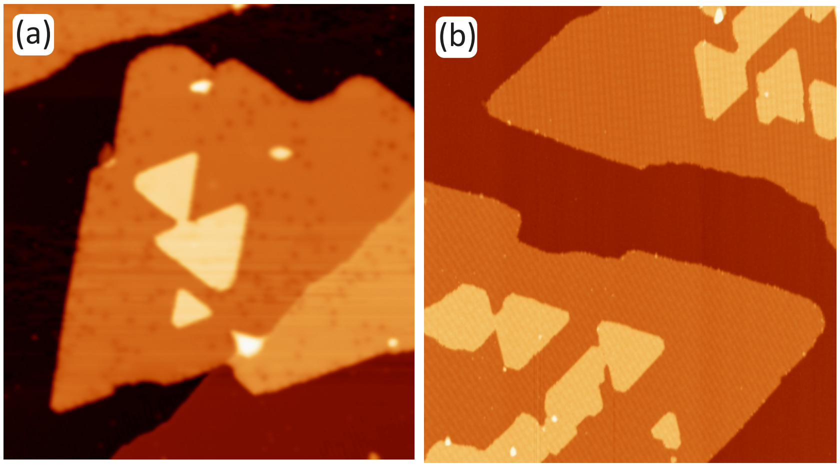

Figure S2. STM topograph of $MnBr_2$ grown at 400 K on (a) Gr/Ir(111) and (b) Gr(Ir(110). (b) is identical to Figure 1(b) of the main manuscript and is reproduced here for direct comparison. Tunneling parameters are for (a) V_b = 3.5 V, 20 pA. and for (b) V_b = -2 V, 50 pA. Image size for (a) and (b) is 180 nm × 200 nm.

Supplementary Note 3. Comparison of Gr/Ir(110) with $MnBr_2$/Gr/Ir(110)

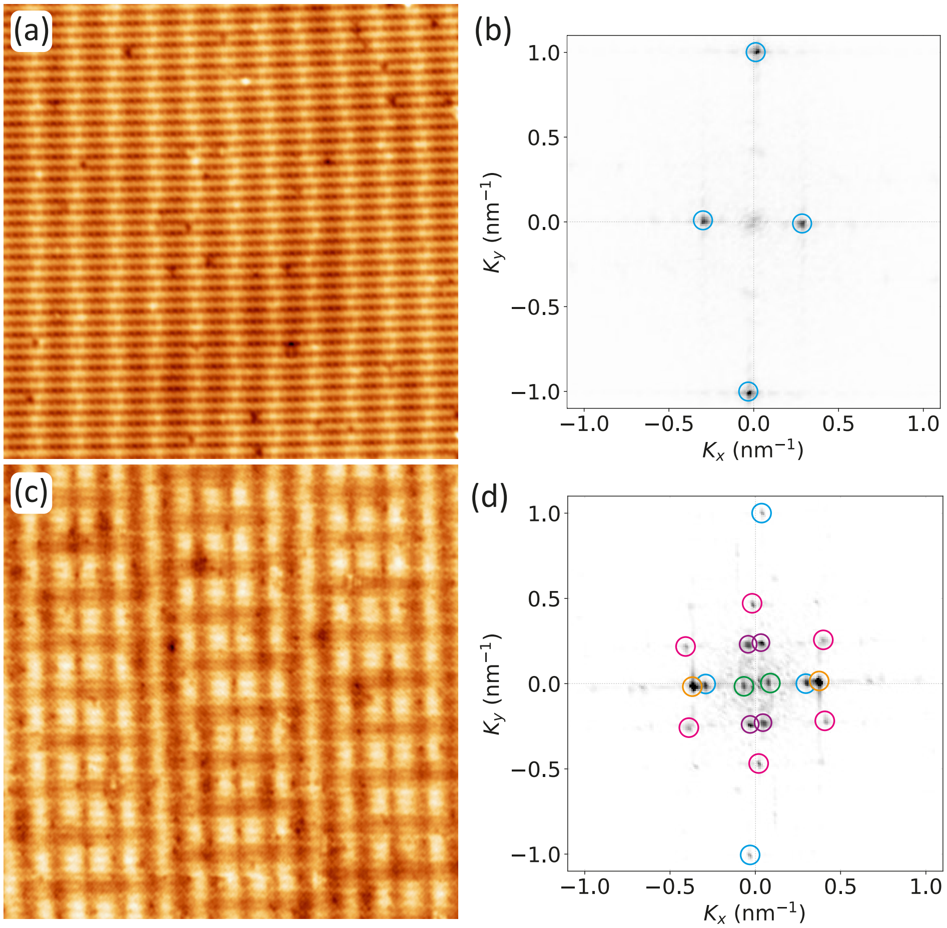

Figure S3. (a) Moiré resolution STM topography of Gr/Ir(110) and (b) its corresponding FFT. (c) Moiré resolution STM topography of $MnBr_2$/Gr/Ir(110) and (c) its corresponding FFT. The Fourier components encircled blue in (b) and (d) are identical. (c) and (d) are Figures 5(a) and (b) of the paper and are reproduced for direct comparison. STM imaging parameters are (a) $V_b =$ -1 V, 100 pA and (c) $V_b =$ -1 V, 50 pA. STM image size is 40 nm $\times$ 40 nm.

Supplementary Note 4. Contour of constant total charge density above Gr/Ir(110)

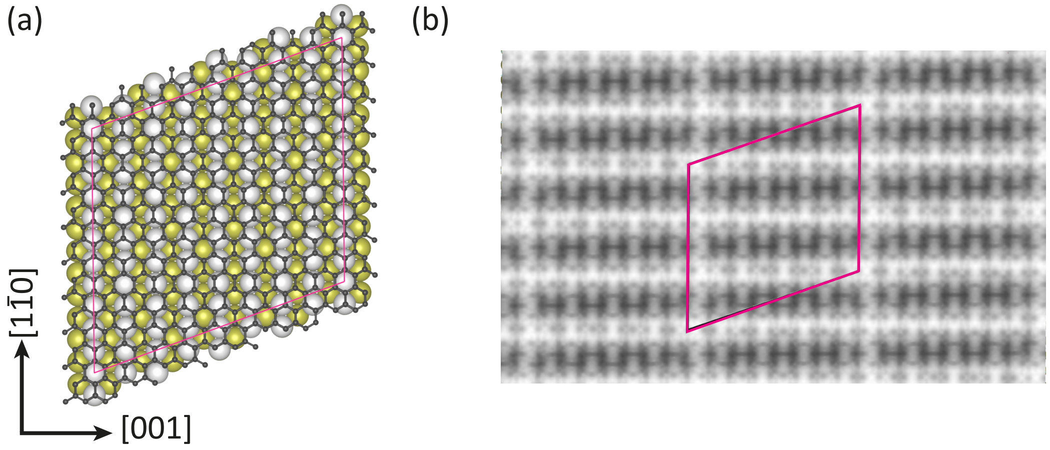

Figure S4. (a) Ball model of atomic arrangement in the supercell. Green gold balls: bottom-layer Ir atoms; white balls: top-layer Ir atoms; dark gray balls: C atoms. A pink rhombus indicates the supercell size in the DFT calculations. (b) The total charge density plotted above the surface for an isosurface value of 2.1×10^{-10} electrons/Å^3. The grayscale encodes the variation in isosurface height. The periodicity of the Ir(110) top layer substrate rows with their 3.84 Å spacing is prominent.

Supplementary Note 5. DFT simulation of $MnBr_2$/Gr/Ir(110)

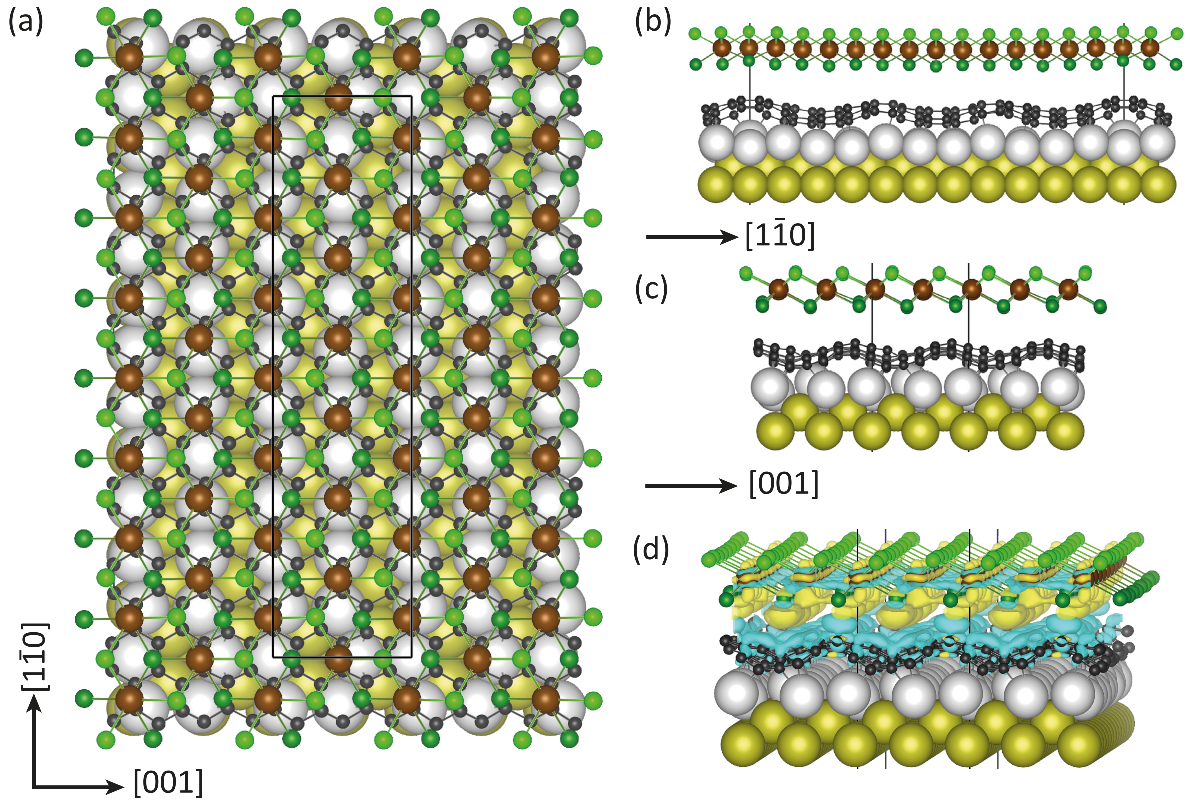

Figure S5. (a) Top view of the relaxed structure of $MnBr_2$ on Gr/Ir(110). The size of supercell is indicated by black rectangle. It is smaller by a factor of ≈ 4 compared to the supercell used to model the Gr/Ir(110) moiré in Figure 6(d) of the main paper. (b),(c) Side views along (b) [001] direction and (c) [1$\bar{1}$0] direction. Black lines indicate boundaries of supercell. (d) The charge density difference is plotted above the surface for an isosurface value 4.9×10^{-8} e/Å^3. Yellow indicates charge density accumulation and blue charge density depletion.

DFT calculations for the entire super-moiré unit cell are impossible. Even including the $MnBr_2$ layer in the unit cell as shown in Figure 9(a) is computationally very expensive and practically not feasible. However, to get an insight into the interaction that occurs between $MnBr_2$ and the Gr/Ir(110) substrate we can use a smaller supercell that correctly captures the chemistry at the interface of $MnBr_2$ and Gr/Ir(110). The supercell used in our calculations is indicated by a black rectangle in Figure S5(a) and contains 14 Mn, 28 Br, 84 C, and 66 Ir atoms. The relative lattice parameters with respect to their nominal values are 0.92

for Ir, 0.96 for Gr, and 1.05 for Mn. We note here that the choice of the unit cell implies a compressive stress for Gr/Ir(110) substrate and tensile stress for the $MnBr_2$ layer.

Our calculations show that the distances between the C atoms of Gr and the lower Br atoms of $MnBr_2$ vary between 2.9 Å and 4.3 Å, as is typical for van der Waals binding [see Figures S5(b) and (c)]. The adsorption energy for the unit cell used in the calculation is 2.86 eV, corresponding to 14 meV/Å^2 or 34 meV per carbon atom. As a note, the interaction occurring between the graphene layers in graphite is 61 meV per carbon atom [1]. Furthermore, we found that there is practically no hybridization between Br and C atoms. We also investigated the charge transfer that occurs at the interface. Figures S5(d) plots the charge density difference when the two parts of the system, Gr/Ir(110) and $MnBr_2$, are joined. Isosurfaces for charge accumulation and charge depletion are colored yellow and blue, respectively. For the entire unit cell 0.092 of an electron is transferred from graphene to $MnBr_2$, mainly to the lower bromine layer in contact with Gr. Therefore, we can also rule out any significant ionic contribution to the binding between $MnBr_2$ and Gr/Ir(110). As a consequence, our calculations demonstrate that the $MnBr_2$ binds the Gr/Ir(110) surface only via rather weak van der Waals interactions.

[1] R. Zacharia, H. Ulbricht, and T. Hertel, Physical Review B **69**, 155406 (2004).
